# Association Between Admission Neutrophil Count and Preoperative Deep Vein Thrombosis Risk in Older Patients with Hip Fracture: A Retrospective Cohort Study

**DOI:** 10.3390/jcm15197363

**Published:** 2026-09-22

**Authors:** Yi-Fan Feng, Wen-Si Qiang, Tian Hu, Yi-Lun Wu, Zhi-Min Hou, Bin-Fei Zhang

**Affiliations:** 1 Department of Ultrasound Medicine, Honghui Hospital, Xi’an Jiaotong University, No. 555 Youyi East Road, Beilin District, Xi’an 710054, China; 2 Department of Joint Surgery, Honghui Hospital, Xi’an Jiaotong University, Xi’an 710054, China

**Keywords:** neutrophil count, deep venous thrombosis, elder, hip fracture, cohort study

## Abstract

**Objective:** This study investigated the association between admission neutrophil count and the risk of preoperative deep vein thrombosis (DVT) in older patients with hip fractures. **Methods:** We conducted a retrospective cohort study of patients aged ≥65 years with hip fractures between January 2015 and September 2019. The primary outcome was the presence of preoperative DVT diagnosed by color Doppler ultrasonography. We used multivariable logistic regression models to assess the association. We examined nonlinearity using smooth curve fitting and threshold effect analysis. **Results:** Among the 1807 patients, 571 (31.6%) developed preoperative DVT. We identified a nonlinear relationship with a threshold effect. The inflection point for neutrophil count was 3.17 × 10^9^/L. Below this threshold, no significant association with DVT risk was observed (OR = 1.90, 95% CI: 0.90–3.99, *p* = 0.092). Interestingly, above this threshold, each 1 × 10^9^/L increase in neutrophil count was associated with 8% lower odds of preoperative DVT (OR = 0.92, 95% CI: 0.89–0.96, *p* < 0.001). **Conclusions:** In older hip fracture patients, admission neutrophil count exhibits a curvilinear association with preoperative DVT risk with a threshold effect at 3.17 × 10^9^/L. This data-driven threshold finding is exploratory and hypothesis-generating. It may support subsequent external validation and mechanistic research.

## 1. Introduction

Hip fracture in older adults constitutes a severe traumatic event. As the global population ages, its incidence continues to rise, making it a significant public health concern [1]. These patients not only face the pain and functional loss caused by the fracture itself but, due to their advanced age, frequent comorbidities, and forced immobilization, are also at extremely high risk for venous thromboembolism (VTE), particularly lower extremity deep vein thrombosis (DVT) [2]. Studies indicate that without prophylaxis, the incidence of DVT following older hip fracture can be as high as 20% [3] to 31% [4]. Preoperative DVT carries substantial risks; it is not only a local cause of postoperative pain and swelling but also a primary source of fatal pulmonary embolism [5,6]. It severely impacts postoperative recovery, prolongs hospital stays, increases healthcare burdens [7], and significantly elevates patient morbidity and mortality rates. Therefore, an in-depth investigation into the predisposing factors and mechanisms of preoperative DVT in older hip fracture patients is of critical clinical importance for implementing precise prevention strategies and improving patient prognosis.

Under the dual impact of fracture trauma and subsequent surgical intervention, the body rapidly initiates a series of complex pathophysiological responses, among which the activation of a systemic inflammatory response is a core feature [8]. Neutrophils, as pioneer cells of innate immunity, play a key role in this process [9]. After trauma, neutrophil counts often rise rapidly as neutrophils release large quantities of reactive oxygen species, proteolytic enzymes, and inflammatory mediators, and form structures such as neutrophil extracellular traps (NETs) [10]. This not only damages surrounding tissues through direct action or amplified pro-inflammatory responses [11], but may also profoundly affect vascular endothelial function and the balance of the coagulation–fibrinolysis system [12,13]. Recent studies suggest that activated neutrophils can initiate and propagate thrombosis through multiple pathways, including inducing endothelial injury, releasing procoagulant substances, promoting platelet aggregation, and contributing to a hypercoagulable state [14,15]. In older hip fracture patients, an intrinsic link may exist between intense, sustained inflammatory responses and the risk of venous thrombosis [16]. Peripheral blood neutrophil count, as a core inflammatory marker, may serve as a convenient, objective biomarker reflecting a pro-thrombotic state and its association with DVT.

However, current research on predicting preoperative DVT risk in older hip fracture patients predominantly focuses on traditional clinical risk assessment models, D-dimer levels, or specific coagulation parameters. Although “immunothrombosis” has become a cutting-edge focus [17,18], clinical studies specifically investigating the association between neutrophil count at hospital admission and the risk of preoperative DVT in this population remain insufficient. Exploring this relationship is significant for two reasons. Firstly, it helps deepen the understanding of the pathological mechanisms of post-fracture thrombosis from an inflammatory perspective. Second, if neutrophil count proves to be an effective predictor, it could provide clinicians with a simple, rapid, and cost-effective tool for generating hypotheses about patient subgroups. Identifying high-risk patients could enable individualized, preemptive anticoagulant prophylaxis, optimize the allocation of medical resources, and ultimately provide a new scientific basis and practical direction for reducing the incidence of preoperative DVT and improving the overall prognosis of older hip fracture patients. Based on this background, we hypothesized that a nonlinear threshold association, rather than a simple linear correlation, exists between admission neutrophil count and preoperative DVT status in older hip fracture patients. This study aimed to explore this observational association and generate hypotheses for further verification. Therefore, this retrospective cohort study systematically evaluated the correlation between admission neutrophil count and preoperative DVT in older patients with hip fractures.

## 2. Materials and Methods

### 2.1. Study Design

This study was based on a retrospective chart review of electronic medical records in our hospital. We retrospectively analyzed all patients’ demographic and clinical data from this database.

This retrospective cohort study included older patients with hip fractures in Xi’an Honghui Hospital between January 2015 and September 2019. The Ethics Committee of Honghui Hospital approved the study (Approval No. 202201009), and the hospital’s Institutional Review Board waived the requirement for informed consent because of the retrospective design.

### 2.2. Participants

We extracted patients’ demographic characteristics and clinical information from their original medical records. The study inclusion criteria were defined as follows: (1) age of 65 years or older; (2) a confirmed diagnosis of femoral neck, intertrochanteric, or subtrochanteric fracture via X-ray or computed tomography; (3) receipt of either surgical or conservative treatment at the hospital; (4) complete availability of in-hospital clinical data. Patients with the following conditions were excluded: (1) active infection at admission (confirmed by clinical symptoms, laboratory tests, and imaging); (2) history of hematological malignancy; (3) chronic inflammatory diseases (including rheumatoid arthritis, systemic lupus erythematosus, inflammatory bowel disease, etc.); (4) immunosuppressive therapy within 3 months before admission; (5) previous history of venous thromboembolism; (6) history of antithrombotic medication use.

### 2.3. Exposure Definition

The exposure was admission neutrophil count, defined as the absolute neutrophil count measured in peripheral venous blood samples collected within 2 h after admission. All blood samples were tested in our hospital’s central laboratory using an automatic hematology analyzer, with a laboratory reference range of (2–7.7) × 10^9^/L.

### 2.4. Perioperative Thromboprophylaxis Protocol

Preoperative evaluations for all patients included routine blood tests and ultrasonographic examinations. For intertrochanteric fractures, the primary therapeutic approach was closed or open reduction and internal fixation (CRIF/ORIF) with a proximal femoral nail anti-rotation device. In the case of femoral neck fractures, hemiarthroplasty (HA) or total hip arthroplasty (THA) was selected as the surgical option based on the patients’ age.

Prophylaxis for DVT was initiated at admission. All patients received standardized thromboprophylaxis after admission. Low-molecular-weight heparin (Fraxiparine; Glaxo Wellcome Production, GlaxoSmithKline, Notre-Dame-de-Bondeville, France) was subcutaneously administered once daily at a weight-adjusted conventional dose. It was initiated within 24 h after admission, stopped 12 h before surgery, and resumed 12 h after surgery. Contraindications included active bleeding, severe coagulation dysfunction, and hypersensitivity to heparin preparations. All eligible patients received mechanical prophylaxis with intermittent pneumatic compression for 20 min twice daily, with good adherence.

The temporal sequence of key procedures was as follows: (1) peripheral venous blood samples for neutrophil count measurement were collected immediately after admission; (2) low-molecular-weight heparin prophylaxis was initiated within 24 h of admission; and (3) the first lower extremity venous ultrasound examination was performed within 24 h of admission. Of note, the ultrasound examination was consistently completed before the initiation of low-molecular-weight heparin, ensuring that the diagnosis of DVT was not influenced by anticoagulant administration.

### 2.5. Ultrasonography Examination Protocol for DVT in Patients

Regarding the timing of ultrasonography, all enrolled patients underwent their first lower extremity venous color Doppler ultrasonography within 24 h of admission. The primary outcome of this study was preoperative DVT. This study implemented systematic and universal preoperative ultrasound screening for all enrolled patients, regardless of whether they presented with DVT-related clinical symptoms such as lower extremity swelling, pain, or increased skin temperature. Attending physicians or above performed all ultrasound examinations, with at least 5 years of experience diagnosing peripheral vascular diseases, and two senior associate chief physicians or above reviewed and confirmed the diagnostic results. The internationally accepted ultrasound diagnostic criteria were adopted for DVT diagnosis, and a definite diagnosis could be made if any one of the following criteria was satisfied: the venous lumen could not be completely compressed by the ultrasound probe; solid hypoechoic or isoechoic thrombus signals were detected within the venous lumen; color Doppler flow imaging showed filling defects or absent flow signals in the venous lumen; or pulse Doppler revealed no significant enhancement of the flow spectrum after compression of the distal limbs. All ultrasound examinations routinely explored all venous segments of bilateral lower extremities, including: proximal deep veins (inferior vena cava, common iliac vein, external iliac vein, common femoral vein, superficial femoral vein, popliteal vein); distal deep veins (anterior tibial vein, posterior tibial vein, peroneal vein); calf intramuscular veins. Clinical sonographers independent of the research team performed all ultrasound examinations. These sonographers did not participate in this retrospective study and were completely unaware of the patients’ laboratory data, including neutrophil counts. The diagnosis of DVT was made solely based on ultrasound imaging findings. Two senior associate chief physicians or above subsequently reviewed and confirmed the diagnostic results.

### 2.6. Statistical Analysis

Continuous variables were presented as mean ± standard deviation for those conforming to a Gaussian distribution, and as median (range) for skewed distributions. Categorical variables were expressed as frequencies with corresponding percentages. Differences in preoperative neutrophil levels across groups were compared using the chi-square test for categorical variables, one-way analysis of variance (ANOVA) for normally distributed continuous variables, and the Kruskal–Wallis H test for skewed continuous variables. We conducted univariate and multivariate logistic regression analyses to examine the association between preoperative neutrophil levels and DVT. We selected confounders based on univariate analysis (*p* < 0.1) and previously reported factors. We included variables significantly associated with DVT in univariate analysis and/or consistently reported in the literature as potential confounders in the multivariable logistic regression model. The final adjusted model included: sex, associated injuries, dementia, fracture classification, and time to admission. The covariates used for adjustment, including sex, associated injuries, dementia, fracture classification, and time to admission, had no missing values. Because thromboprophylaxis was uniformly applied to all patients under a standardized institutional protocol, it was not included as a covariate in the multivariable models to avoid overadjustment.

Given the common concern that logistic regression may be inadequate for modeling nonlinear associations, we used a generalized additive model with smooth curve fitting (via penalized splines) to examine the nonlinear relationship between preoperative neutrophil levels and DVT. We used a generalized additive model with penalized splines to fit the nonlinear association. We automatically selected the smoothing parameter using Restricted Maximum Likelihood, minimizing the Akaike Information Criterion to balance model fit and parsimony. We used the gam function in the mgcv package in R with penalized splines (bs = “tp”). We selected the smoothing parameter using REML. We set the number of knots to 3 by default and ran sensitivity analyses with 2–5 knots to confirm stability. We formally assessed nonlinearity using the log-likelihood ratio test comparing the linear and nonlinear models.

If nonlinearity was identified, we first used a recursive algorithm to calculate the inflection point, then constructed a two-piecewise logistic regression model for the data segments on either side of this point. We used the bootstrap recursive algorithm to identify the inflection point. This algorithm iteratively computes the model’s log-likelihood for different cut-off points and selects the cut-off point with the maximum log-likelihood as the optimal inflection point. Meanwhile, we used the Bootstrap method with 1000 repeated samples to estimate the 95% confidence interval (95%CI) of the inflection point. We conducted sensitivity analyses with 2–5 knots for the smoothing spline to assess the stability of the fitted curve, and the nonlinear pattern remained stable across these specifications. The inflection point was data-driven and should be regarded as exploratory and hypothesis-generating rather than a validated clinical cutoff value.

The primary goal of this study was to estimate the total effect of admission neutrophil count on preoperative DVT risk in older hip fracture patients, rather than the direct effect. Therefore, we presented the core results based on the minimal confounder set, and used the adjusted model including potential mediator variables only for sensitivity analysis to verify the stability of the results.

All statistical analyses were implemented using R software version 4.5.3 (http://www.R-project.org, accessed on 26 January 2026, R Foundation for Statistical Computing, Vienna, Austria) and EmpowerStats software (http://www.empowerstats.com, accessed on 26 January 2026, X&Y Solutions Inc., Boston, MA, USA, version 5.0). Odds ratios (ORs) and their corresponding 95%CIs were computed for all regression analyses. We considered *p* < 0.05 statistically significant for all tests.

## 3. Results

### 3.1. Patient Characteristics

Of the consecutive participants with hip fractures between Jan 2015 and Sep 2019, we included 1807 who met the study criteria. Figure 1 shows the study population flow diagram. The preoperative neutrophil count was 6.89 ± 2.76 (×10^9^). A total of 571 (31.6%) patients were diagnosed with preoperative DVT. We divided neutrophil concentration into three groups: low (0.92–5.43 × 10^9^/L), medium (5.44–7.69 × 10^9^/L), and high (7.71–23.09 × 10^9^/L). We determined these cutoffs based on tertiles of the neutrophil count distribution in the entire cohort. Table 1 lists the demographic and clinical characteristics of all patients and their comorbidities, factors associated with injuries, and treatment baselines. We also compared baseline characteristics between included (n = 1807) and excluded (n = 1348) patients, as shown in Supplementary Table S1.

The median time from injury to admission was 11 h. All patients underwent peripheral venous blood sampling immediately after admission according to the standardized institutional protocol, and the first lower extremity venous ultrasound was performed within 24 h of admission. However, the electronic medical record system did not record the exact times of blood sampling and ultrasound examination; therefore, we could not calculate the precise admission-to-sampling and admission-to-ultrasound intervals.

### 3.2. Univariate Analysis of the Association Between Variables and DVT

To identify potential confounding factors and the relationship between variables and DVT, we performed a univariate analysis (Table 2). Based on the *p* < 0.1 criterion and previously reported factors, we included the following variables in the multivariate logistic regression: sex, associated injuries, dementia, fracture classification, and time to admission.

### 3.3. Multivariate Analysis of Preoperative Neutrophil and DVT

We used two models (Table 3) to build the association between preoperative neutrophil levels and DVT. When neutrophil count was included as a continuous variable, logistic regression analysis showed that in the adjusted model, each 1 × 10^9^/L increase in admission neutrophil count was associated with an approximately 7% reduction in the odds of preoperative DVT (OR = 0.93, 95% CI: 0.89–0.97, *p* < 0.001). When neutrophil count was included as a categorical variable in tertiles, the odds of DVT were significantly lower in the high tertile than in the low tertile, with a significant linear trend across the three tertiles (*p* for trend < 0.05). However, we observed that the association was attenuated in the high-level subgroup, suggesting the relationship may not be simply linear. Therefore, we further refined the nonlinear relationship using smooth curve fitting and threshold-effect analysis, which showed that the association could be divided into two segments with different slopes below and above the inflection point.

### 3.4. Curve Fitting and Analysis of the Threshold Effect

As shown in Figure 2, there was a curved association between preoperative neutrophil level and DVT after adjusting for confounding factors. We compared two fitting models to explain this association (Table 4). Interestingly, we observed an inflection point at 3.17 × 10^9^ in the threshold effect, with *p* = 0.045 for the log-likelihood ratio test. The 95%CI for the inflection point was 3.07 to 4.21. A neutrophil count of <3.17 × 10^9^ was not associated with DVT (OR = 1.90, 95% CI: 0.90–3.99, *p* = 0.092). At a neutrophil count > 3.17 × 10^9^, the count was associated with a decrease in DVT risk (OR = 0.92, 95% CI: 0.89–0.96, *p* < 0.001), which means that a 1 × 10^9^ increase in neutrophil count was associated with 8% lower odds of preoperative DVT after hip fracture. The inflection point was data-driven, and it should be regarded as exploratory and hypothesis-generating rather than a validated clinical cutoff value.

### 3.5. In Exploratory Subgroup Analysis of Proximal and Distal DVT

Among the 571 patients with preoperative DVT, 89 (15.6%) had proximal DVT and 482 (84.4%) had distal DVT. Subgroup analysis showed a significant nonlinear threshold association between admission neutrophil count and distal DVT risk (inflection point 3.21 × 10^9^/L, consistent with the main analysis). When the neutrophil count was above the inflection point, each 1 × 10^9^/L increase was associated with a 9% reduction in distal DVT (OR = 0.91, 95% CI: 0.87–0.95, *p* < 0.001). For proximal DVT, no significant nonlinear association was observed; there was no linear association between neutrophil count and DVT (OR = 0.97, 95% CI: 0.93–1.01, *p* = 0.128). However, proximal DVT events were few, and this analysis was underpowered. These subgroup findings are exploratory and hypothesis-generating only. The difference in statistical significance between proximal and distal DVT should not be interpreted as evidence of a biological difference, as it may reflect differences in event numbers and statistical power.

## 4. Discussion

Through a retrospective cohort analysis, this study systematically investigated the association between admission neutrophil count and the risk of preoperative DVT in older hip fracture patients. Our findings reveal that this relationship is not linear but exhibits a significant curvilinear association with a threshold effect. Specifically, when the admission neutrophil count was below 3.17 × 10^9^/L, its level showed no significant association with DVT risk. However, above this threshold, higher neutrophil counts were inversely associated with DVT risk; for every 1 × 10^9^/L increment, the odds of DVT decreased by about 8%. This finding challenges the conventional notion that “a higher degree of inflammation correlates with a greater thrombotic risk,” suggesting that a moderate early inflammatory response may be associated with a specific clinical context: older hip fracture. Our research not only provides new clinical evidence for understanding post-fracture immune-thrombotic interactions but, more importantly, identifies an exploratory data-driven threshold that warrants external validation (3.17 × 10^9^/L). This exploratory nonlinear association provides new hypothesis-generating evidence for understanding immune-thrombosis interaction after hip fracture and warrants further external validation in independent cohorts.

Our findings both resonate with and importantly diverge from numerous previous studies exploring the relationship between inflammation and venous thrombosis. Extensive basic and clinical research supports the theory of “immunothrombosis,” in which the overactivation of inflammatory cells, such as neutrophils, promotes thrombus formation through mechanisms including the release of neutrophil extracellular traps (NETs) and pro-inflammatory factors [14,19,20,21]. In major orthopedic surgeries (e.g., joint replacement) or in critically ill patients with severe infections, an elevated neutrophil count is often considered a risk factor for thrombotic events [22,23]. However, these studies predominantly focus on the postoperative phase or patients with significant infections, whose inflammatory context differs from that of isolated traumatic fractures in the older. Liu et al. found that levels of Citrullinated Histone H3, a marker of NETs, were significantly higher in patients who developed DVT after traumatic fracture compared to those who did not, with another marker, cfDNA, also markedly increasing following DVT formation [24]. Similarly, Li et al. reported that preoperative circulating NETs levels were higher in lumbar fracture patients who developed postoperative DVT compared to those who did not [25].

Furthermore, other neutrophil-related ratio indicators also suggest an involvement of neutrophils in DVT [26,27]. While these studies indicate that neutrophils may participate in DVT formation, the precise nature of the relationship between neutrophil levels and DVT risk remains unclear. Our study is the first to conduct an in-depth analysis of neutrophil count at early preoperative hospital admission in a large cohort of older hip fracture patients, revealing a unique nonlinear relationship with DVT risk. The finding that an elevated admission neutrophil count was associated with reduced preoperative DVT risk in older hip fracture patients clearly contradicts the classical ‘immunothrombosis’ theory, which holds that neutrophil activation promotes thrombosis. This paradox needs to be interpreted in combination with the special pathophysiological state of older trauma patients. This suggests that during the initial phase of traumatic stress, the nature and consequences of the body’s inflammatory response may be stage-specific. Our research validates and precisely quantifies this nonlinear pattern, particularly by defining a concrete threshold within a more homogeneous, higher-risk population of older hip fracture patients. This approach offers greater clinical guidance than analyses based on simple linear associations. Therefore, by focusing on a specific population and time phase and employing a more refined statistical model (threshold effect analysis), this study deepens understanding of the complex relationship between early traumatic inflammatory response and thrombosis risk, addressing a gap in this field.

The following mechanistic explanations are speculative and hypothesis-generating, without direct experimental evidence from the present observational study. The observed nonlinear relationship may be speculatively attributed to neutrophil functional heterogeneity and the bidirectional regulation of inflammation [28,29,30]. Below 3.17 × 10^9^/L, a low neutrophil count may reflect immunosenescence or inadequate bone marrow reserve in older patients, leading to poor tissue damage clearance and potentially triggering coagulation and increasing DVT risk. Above the threshold, a moderately elevated count indicates an effective innate immune response that clears debris and stabilizes the microenvironment, which may paradoxically protect against thrombosis. However, extremely high counts may signal excessive inflammation or infection, where pro-thrombotic effects could dominate again—consistent with the attenuated association in the high-level subgroup. Alternative explanations should also be considered. First, time from injury to admission varied considerably and may influence both neutrophil count and DVT status. Second, immunosenescence in older adults may alter the relationship between peripheral neutrophil count and functional immune status. Third, unmeasured comorbidities and medications may contribute to residual confounding. Fourth, the possibility of reverse causation—whereby pre-existing DVT influences systemic inflammatory markers—cannot be excluded.

This exploratory finding may offer potential academic implications for future clinical research. As an easily accessible and routine inflammatory indicator, admission neutrophil count could serve as a tentative reference complementary to traditional risk assessment tools such as the Caprini score. This threshold association may support the hypothesis that patients with a relatively low admission neutrophil count below 3.17 × 10^9^/L may have altered susceptibility to preoperative DVT. At present, this observation cannot support immediate clinical decision-making. However, it may support further prospective studies to determine whether this subgroup needs closer monitoring or tailored thromboprophylaxis protocols.

To further address the potential confounding effect of time from injury to admission, we conducted two sensitivity analyses. First, we included this variable as a restricted cubic spline in the logistic regression model to allow for nonlinear adjustment. Second, we restricted the cohort to patients admitted within 72 h of injury (n = 1523, 84.3% of the total cohort). In both sensitivity analyses, the nonlinear association between admission neutrophil count and preoperative DVT remained significant, with inflection points of 3.18 × 10^9^/L and 3.22 × 10^9^/L, respectively, consistent with the primary analysis (3.17 × 10^9^/L). These findings suggest that time from injury to admission is unlikely to be a major confounder in our study.

This study has several strengths. First, it used a retrospective cohort design with systematic, standardized data collection. Second, the relatively large overall sample size provided adequate statistical power for the primary analysis. However, for subgroup analyses, particularly the proximal DVT subgroup (n = 89) and the analysis below the inflection point, statistical power was limited, and the corresponding null findings should be interpreted with caution. Third, we collected and adjusted for a wide range of potential confounders. Fourth, advanced statistical methods (smooth curve fitting and threshold effect analysis) uncovered a complex relationship that traditional linear models might obscure, yielding more robust conclusions.

There were some limitations. First, although we adjusted for key covariates, our confounding control relied partly on univariate screening (*p* < 0.1) and previously reported factors rather than a formal causal framework. Residual confounding therefore cannot be excluded. In addition, we did not collect body mass index because we could not obtain accurate height and weight in some patients due to bed rest or forced positioning, which may further contribute to residual confounding. Second, DVT diagnosis relied on a single preoperative ultrasound performed within 24 h of admission. As no repeat preoperative ultrasound was routinely performed, we could not assess the progression or resolution of DVT before surgery, nor capture DVT that might have developed after the initial ultrasound but before surgery. Third, we analyzed only a single admission neutrophil count, which cannot capture dynamic changes over time. Fourth, as a retrospective observational study, this study can only demonstrate an association, not a causal relationship; neutrophil count is more likely a composite biomarker reflecting traumatic stress and the immune–coagulation network rather than a direct pathogenic driver. Finally, 1348 patients were excluded because of missing ultrasound results or neutrophil counts. Compared with included patients, excluded patients were older and more likely to be male. The missingness was mainly due to ultrasound reports no longer being retrievable from the electronic medical record system (n = 1287) and missing neutrophil count records (n = 47; 2 patients had no blood sample collected and 45 had results not recorded), suggesting that the missingness may be informative rather than completely at random. Because older age may be associated with a higher risk of DVT, whereas male sex in our cohort was associated with a lower risk of DVT, the net direction of potential bias is mixed and cannot be determined with certainty. Nevertheless, this may affect the observed DVT prevalence and association estimates, and the results should be generalized with caution.

## 5. Conclusions

This study demonstrates a significant curvilinear relationship between admission neutrophil count and the risk of preoperative DVT in older patients with hip fracture, with a threshold identified at 3.17 × 10^9^/L. Notably, neutrophil counts above this threshold were independently associated with a decreased risk of DVT. The identified data-driven threshold is exploratory and requires prospective multicenter external validation. These findings show only an observational curvilinear association and may provide a theoretical hypothesis for future research on immune–inflammatory balance and thrombosis after older hip fracture, rather than guiding immediate individualized clinical thromboprophylaxis.

## Figures and Tables

**Figure 1:**
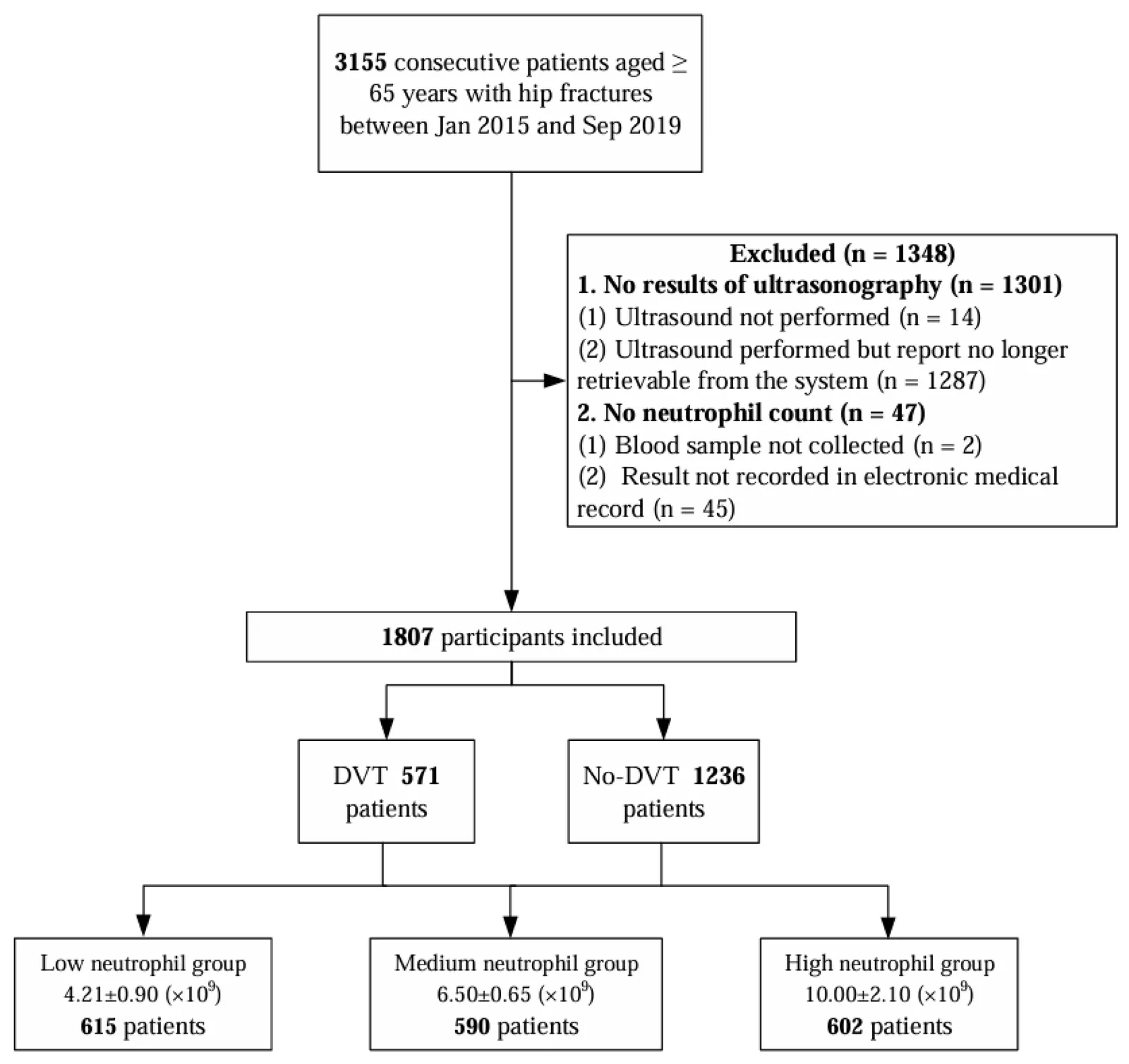
The flow diagram of the study population.

**Figure 2:**
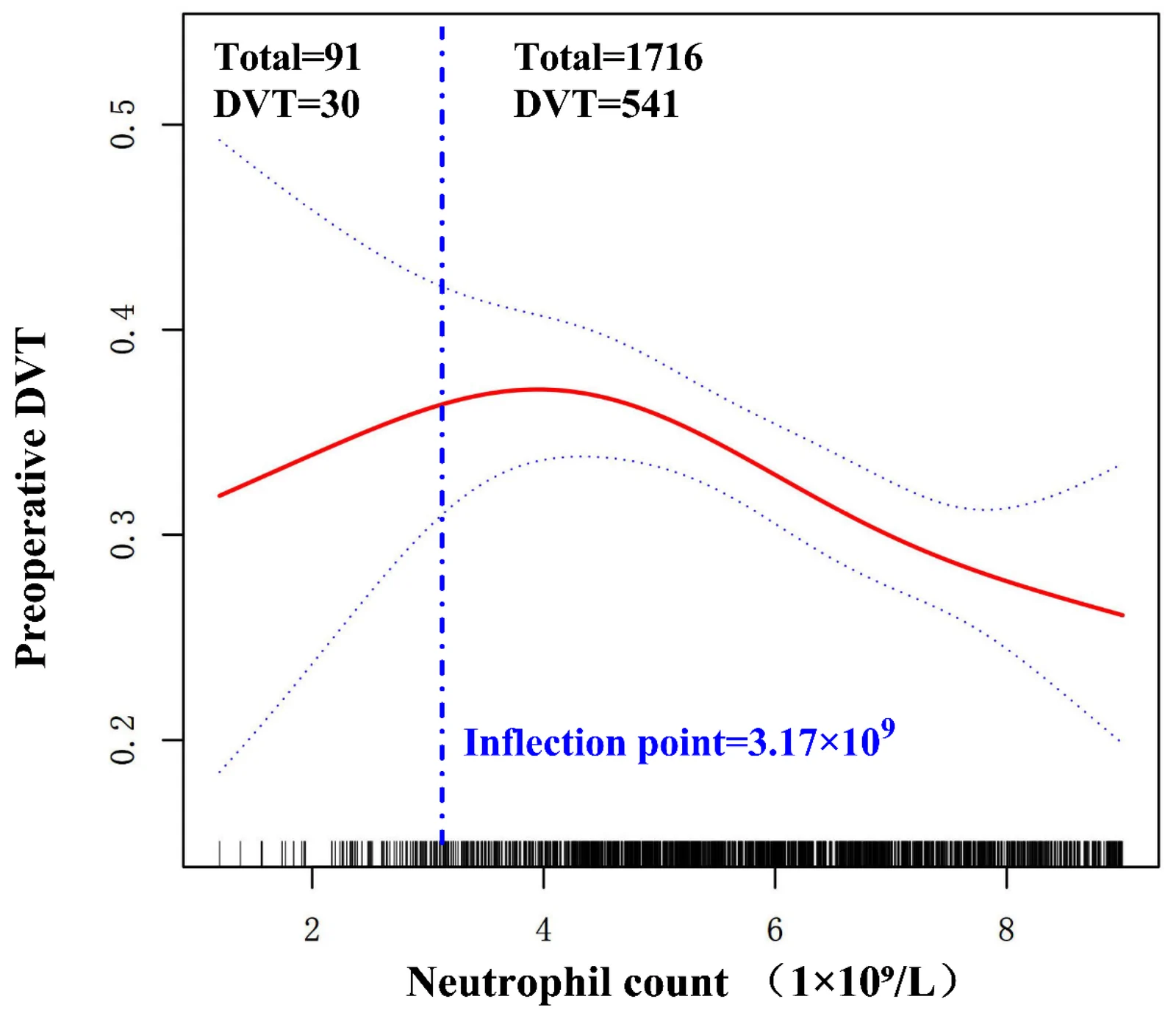
Curve fitting between preoperative neutrophil and DVT. Adjusted for sex, associated injuries, dementia, fracture classification, and time to admission.

**Table 1:** Demographic and clinical characteristics.

Neutrophil Level	Low	Medium	High	*p*-Value	*p*-Value *
N	615	590	602		
Age (y)	79.09 ± 7.05	79.60 ± 6.83	79.66 ± 6.73	0.289	0.180
Sex				0.955	-
Male	181 (29.43%)	178 (30.17%)	181 (30.07%)		
Female	434 (70.57%)	412 (69.83%)	421 (69.93%)		
Neutrophil count (×10^9^)	4.21 ± 0.90	6.50 ± 0.65	10.00 ± 2.10	<0.001	<0.001
Injury mechanism				0.308	-
Falling	592 (96.26%)	569 (96.44%)	584 (97.01%)		
Accident	16 (2.60%)	19 (3.22%)	16 (2.66%)		
Other	7 (1.14%)	2 (0.34%)	2 (0.33%)		
Fracture classification				<0.001	-
Intertrochanteric fracture	336 (54.63%)	359 (60.85%)	411 (68.27%)		
Femoral neck fracture	279 (45.37%)	231 (39.15%)	191 (31.73%)		
aCCI	4.13 ± 1.09	4.23 ± 1.12	4.30 ± 1.09	0.026	0.009
Hypertension				<0.001	-
No	347 (56.42%)	282 (47.80%)	268 (44.52%)		
Yes	268 (43.58%)	308 (52.20%)	334 (55.48%)		
Diabetes				0.298	-
No	500 (81.30%)	478 (81.02%)	470 (78.07%)		
Yes	115 (18.70%)	112 (18.98%)	132 (21.93%)		
CHD				0.002	-
No	329 (53.50%)	279 (47.29%)	263 (43.69%)		
Yes	286 (46.50%)	311 (52.71%)	339 (56.31%)		
Arrhythmia				0.817	-
No	418 (67.97%)	407 (68.98%)	405 (67.28%)		
Yes	197 (32.03%)	183 (31.02%)	197 (32.72%)		
Hemorrhagic stroke				0.405	-
No	601 (97.72%)	581 (98.47%)	594 (98.67%)		
Yes	14 (2.28%)	9 (1.53%)	8 (1.33%)		
Ischemic stroke				0.081	-
No	433 (70.41%)	412 (69.83%)	391 (64.95%)		
Yes	182 (29.59%)	178 (30.17%)	211 (35.05%)		
Cancer				0.191	-
No	595 (96.75%)	573 (97.12%)	592 (98.34%)		
Yes	20 (3.25%)	17 (2.88%)	10 (1.66%)		
Associated injuries				0.453	-
No	578 (93.98%)	547 (92.71%)	555 (92.19%)		
Yes	37 (6.02%)	43 (7.29%)	47 (7.81%)		
Dementia				0.015	-
No	596 (96.91%)	556 (94.24%)	585 (97.18%)		
Yes	19 (3.09%)	34 (5.76%)	17 (2.82%)		
COPD				0.826	-
No	576 (93.66%)	557 (94.41%)	568 (94.35%)		
Yes	39 (6.34%)	33 (5.59%)	34 (5.65%)		
Hepatitis				0.261	-
No	591 (96.10%)	575 (97.46%)	587 (97.51%)		
Yes	24 (3.90%)	15 (2.54%)	15 (2.49%)		
Gastritis				0.464	-
No	604 (98.21%)	580 (98.31%)	596 (99.00%)		
Yes	11 (1.79%)	10 (1.69%)	6 (1.00%)		
Time to admission (h)	48 (1–5040)	10 (1–1440)	5 (1–1440)	<0.001	<0.001
Admission to operation (d)	4 (1–18)	4 (1–34)	4 (1–20)	0.03	0.022
Treatment strategy				<0.001	-
Conservation	28 (4.55%)	40 (6.78%)	41 (6.81%)		
CRIF/ORIF	313 (50.89%)	342 (57.97%)	383 (63.62%)		
HA	254 (41.30%)	196 (33.22%)	170 (28.24%)		
THA	20 (3.25%)	12 (2.03%)	8 (1.33%)		
Creatinine (μmol/L)	66.72 ± 23.11	71.28 ± 33.54	71.02 ± 28.45	0.009	0.011
Urea nitrogen (mmol/L)	6.36 ± 2.45	6.82 ± 2.87	6.89 ± 2.93	0.002	0.002
Estimated glomerular filtration rate (mL/min)	79.47 ± 17.62	76.56 ± 18.65	75.88 ± 18.03	0.008	0.002
Operation time (mins)	90.32 ± 31.57	91.29 ± 35.13	99.17 ± 35.08	<0.001	<0.001
Blood loss (mL)	200 (30–1500)	200 (40–1500)	200 (0–1200)	0.500	0.170
Infusion (mL)	1600 (600–3200)	1600 (600–3200)	1600 (600–3200)	0.001	0.005
Transfusion (U)	0 (0–6)	0 (0–6)	0 (0–6)	0.822	0.724
Stay in hospital (d)	7.15 (3.63–37.02)	7.66 (3.54–25.85)	7.73 (3.54–27.20)	0.022	0.012
Preoperative DVT				0.011	-
No	399 (64.88%)	399 (67.63%)	438 (72.76%)		
Yes	216 (35.12%)	191 (32.37%)	164 (27.24%)		

Mean + SD/N(%)*. p*-value *: For continuous variables, we used the Kruskal–Wallis rank-sum test and Fisher’s exact probability test for count variables with a theoretical number of <10. CHD, coronary heart disease; COPD, chronic obstructive pulmonary disease; aCCI, age-adjusted Charlson comorbidity index; CRIF/ORIF, closed or open reduction and internal fixation; HA, hemiarthroplasty; THA, total hip arthroplasty.

**Table 2:** Effects of factors on DVT measured by univariate analysis.

	Statistics	OR (95% CI)	*p*-Value
Age (y)	79.45 ± 6.87	1.00 (0.99, 1.02)	0.678
Sex			
Male	540 (29.88%)	1	
Female	1267 (70.12%)	1.29 (1.04, 1.61)	0.023
Injury mechanism			
Falling	1745 (96.57%)	1	
Accident	51 (2.82%)	1.09 (0.60, 1.97)	0.770
Other	11 (0.61%)	2.62 (0.80, 8.63)	0.113
Fracture classification			
Intertrochanteric fracture	1106 (61.21%)	1	
Femoral neck fracture	701 (38.79%)	0.68 (0.56, 0.84)	<0.001
Time to admission (h)	11 (1–5040)	1.00 (1.00, 1.00)	0.289
Admission to operation (d)	4.00 (1–34)	1.05 (1.01, 1.10)	0.016
aCCI	4.22 ± 1.10	1.00 (0.91, 1.09)	0.958
Hypertension			
No	897 (49.64%)	1	
Yes	910 (50.36%)	1.14 (0.93, 1.39)	0.208
Diabetes			
No	1448 (80.13%)	1	
Yes	359 (19.87%)	0.96 (0.75, 1.23)	0.757
CHD			
No	871 (48.20%)	1	
Yes	936 (51.80%)	1.09 (0.89, 1.33)	0.405
Arrhythmia			
No	1230 (68.07%)	1	
Yes	577 (31.93%)	1.09 (0.89, 1.35)	0.405
Hemorrhagic stroke			
No	1776 (98.28%)	1	
Yes	31 (1.72%)	1.80 (0.88, 3.68)	0.106
Ischemic stroke			
No	1236 (68.40%)	1	
Yes	571 (31.60%)	0.86 (0.69, 1.07)	0.176
Cancer			
No	1760 (97.40%)	1	
Yes	47 (2.60%)	1.12 (0.61, 2.07)	0.715
Associated injuries			
No	1680 (92.97%)	1	
Yes	127 (7.03%)	1.55 (1.07, 2.24)	0.020
Dementia			
No	1737 (96.13%)	1	
Yes	70 (3.87%)	1.66 (1.02, 2.69)	0.041
COPD			
No	1701 (94.13%)	1	
Yes	106 (5.87%)	0.89 (0.58, 1.37)	0.591
Hepatitis			
No	1753 (97.01%)	1	
Yes	54 (2.99%)	0.68 (0.36, 1.28)	0.230
Gastritis			
No	1780 (98.51%)	1	
Yes	27 (1.49%)	0.49 (0.18, 1.29)	0.149
Treatment strategy			
Conservation	109 (6.03%)	1	
ORIF	1038 (57.44%)	1.07 (0.70, 1.62)	0.760
HA	620 (34.31%)	0.76 (0.49, 1.18)	0.218
THA	40 (2.21%)	0.51 (0.21, 1.21)	0.127
Neutrophil count (×10^9^)	6.89 ± 2.76	0.95 (0.92, 0.99)	0.014
Creatinine (μmol/L)	69.65 ± 28.69	1.00 (1.00, 1.00)	0.743
Urea nitrogen (mmol/L)	6.69 ± 2.77	1.03 (0.99, 1.07)	0.115
Estimated glomerular filtration rate (mL/min)	77.35 ± 18.15	1.00 (0.99, 1.01)	0.940
Operation time (mins)	93.56 ± 34.13	1.00 (1.00, 1.01)	0.140
Blood loss (mL)	200 (0–1500)	1.00 (1.00, 1.00)	0.225
Infusion (mL)	1600 (600–3200)	1.00 (1.00, 1.00)	0.718
Transfusion (U)	0 (0–6)	1.13 (1.04, 1.23)	0.003
Stay in hospital (d)	7.63 (3.54–37.02)	1.03 (1.00, 1.06)	0.054

CHD, coronary heart disease; COPD, chronic obstructive pulmonary disease; aCCI, age-adjusted Charlson comorbidity index; CRIF/ORIF, closed or open reduction and internal fixation; HA, hemiarthroplasty; THA, total hip arthroplasty.

**Table 3:** Univariate and multivariate results by logistic regression.

Exposure	Crude Model	Adjusted Model
Neutrophil count (×10^9^)	0.95 (0.92, 0.99) 0.014	0.95 (0.91, 0.98) 0.006
Neutrophil level		
Low	1	1
Medium	0.88 (0.70, 1.12) 0.313	0.86 (0.67, 1.10) 0.227
High	0.69 (0.54, 0.88) 0.003	0.65 (0.51, 0.85) 0.001
*p* for trend	0.003	0.001

Data in table: OR (95% CI) *p*-value. Outcome variable: DVT. Exposed variable: neutrophil count. The adjusted model included sex, associated injuries, dementia, fracture classification, and time to admission. Number of observations: 1807 and 1807 in the crude model and adjusted model, respectively.

**Table 4:** Nonlinearity of preoperative neutrophil and DVT.

Outcome	Preoperative DVT
Fitting a model by standard linear regression	0.93 (0.89, 0.97) <0.001
Fitting a model by two-piecewise linear regression	
Inflection point	3.17
95%CI	3.07, 4.21
<Inflection point	1.90 (0.90, 3.99) 0.092
>Inflection point	0.92 (0.89, 0.96) <0.001
*p* for log-likelihood ratio test	0.045

Adjusted for sex, associated injuries, dementia, fracture classification, and time to admission.

## Data Availability

The work described has not been published before (except in the form of an abstract or as part of a published lecture, review, or thesis); it is not under consideration for publication elsewhere, and all co-authors have approved its publication. Xi’an Honghui Hospital implemented the data collection. According to relevant regulations, the data cannot be shared but can be requested from the corresponding author.

## Supplementary Materials

The following supporting information can be downloaded at: https://www.mdpi.com/article/10.3390/jcm15197363/s1. Table S1: The Demographic and clinical characteristics between excluded and included patients.
