# Supplementary material for: Association Between Admission Neutrophil Count and Preoperative Deep Vein Thrombosis Risk in Older Patients with Hip Fracture: A Retrospective Cohort Study
Source: J Clin Med. 2026 Sep 22;15(19):7363. doi: 10.3390/jcm15197363 (PMC13665214; doi:10.3390/jcm15197363)
Supplement: Supplementary file 1 [file jcm-15-07363-s001.zip › jcm-4511650-supplementary.pdf]

Table S1. The Demographic and clinical characteristics between excluded and included patients.

	Excluded patients	Included patients	Standardize diff.	*P*-value	*P*-value*
N	1348	1807			
Age (y)	80.22±6.47	79.45±6.87	0.12 (0.05, 0.19)	0.001	0.002
Sex			0.13 (0.05, 0.20)	<0.001	-
Male	482 (35.76%)	540 (29.88%)			
Female	866 (64.24%)	1267 (70.12%)			
Injury mechanism			0.06 (-0.01, 0.13)	0.258	-
Falling	1315 (97.55%)	1745 (96.57%)			
Accident	26 (1.93%)	51 (2.82%)			
Other	7 (0.52%)	11 (0.61%)			
Fracture classification			0.85 (0.77, 0.92)	<0.001	-
Intertrochanteric fracture	1264 (93.77%)	1106 (61.21%)			

Femoral neck fracture	84 (6.23%)	701 (38.79%)			
aCCI	4.28±1.05	4.22±1.10	0.05 (-0.02, 0.12)	0.144	0.124
Hypertension			0.05 (-0.02, 0.13)	0.129	-
No	706 (52.37%)	897 (49.64%)			
Yes	642 (47.63%)	910 (50.36%)			
Diabetes			0.01 (-0.07, 0.08)	0.884	-
No	1083 (80.34%)	1448 (80.13%)			
Yes	265 (19.66%)	359 (19.87%)			
CHD			0.09 (0.02, 0.16)	0.012	-
No	589 (43.69%)	871 (48.20%)			
Yes	759 (56.31%)	936 (51.80%)			
Arrhythmia			0.14 (0.07, 0.21)	<0.001	-
No	825 (61.20%)	1230 (68.07%)			

Yes	523 (38.80%)	577 (31.93%)			
Hemorrhagic stroke			0.08 (0.01, 0.15)	0.026	-
No	1309 (97.11%)	1776 (98.28%)			
Yes	39 (2.89%)	31 (1.72%)			
Ischemic stroke			0.12 (0.05, 0.19)	0.001	-
No	993 (73.66%)	1236 (68.40%)			
Yes	355 (26.34%)	571 (31.60%)			
Cancer			0.05 (-0.02, 0.12)	0.148	-
No	1301 (96.51%)	1760 (97.40%)			
Yes	47 (3.49%)	47 (2.60%)			
Associated injuries			0.02 (-0.05, 0.09)	0.513	-
No	1245 (92.36%)	1680 (92.97%)			
Yes	103 (7.64%)	127 (7.03%)			

Dementia			0.01 (-0.06, 0.08)	0.811	-
No	1298 (96.29%)	1737 (96.13%)			
Yes	50 (3.71%)	70 (3.87%)			
COPD			0.09 (0.02, 0.16)	0.012	-
No	1238 (91.84%)	1701 (94.13%)			
Yes	110 (8.16%)	106 (5.87%)			
Hepatitis			0.02 (-0.05, 0.09)	0.501	-
No	1302 (96.59%)	1753 (97.01%)			
Yes	46 (3.41%)	54 (2.99%)			
Gastritis			0.05 (-0.02, 0.12)	0.167	-
No	1319 (97.85%)	1780 (98.51%)			
Yes	29 (2.15%)	27 (1.49%)			
Neutrophil count (10^9)	6.94±2.82	6.89±2.76	0.02 (-0.05, 0.09)	0.579	0.526

Time to admission (h)	86.05±228.69	84.02±256.56	0.01 (-0.06, 0.08)	0.818	0.162
Time to operation (d)	4.47±2.72	4.15±2.42	0.12 (0.05, 0.20)	0.001	<0.001
Operation time (mins)	92.16±37.91	93.56±34.13	0.04 (-0.04, 0.11)	0.309	0.023
Blood loss (mL)	244.66±158.31	236.92±149.01	0.05 (-0.03, 0.13)	0.193	0.393
Transfusion (U)	1.43±1.22	0.93±1.21	0.41 (0.33, 0.49)	<0.001	<0.001
Infusion (mL)	1509.46±390.52	1587.00±380.91	0.20 (0.12, 0.28)	<0.001	<0.001
Stay in hospital (d)	9.84±4.13	8.25±3.33	0.42 (0.35, 0.49)	<0.001	<0.001
Treatment strategy			0.78 (0.70, 0.85)	<0.001	-
Conservation	207 (15.36%)	109 (6.03%)			
ORIF	1041 (77.23%)	1038 (57.44%)			
HA	96 (7.12%)	620 (34.31%)			
THA	4 (0.30%)	40 (2.21%)			

P-value*: For continuous variables, we used the Kruskal-Wallis rank-sum test and Fisher's exact probability test for count variables with a theoretical number of <10.

CHD, coronary heart disease; **COPD**, chronic obstructive pulmonary disease; **aCCI**. age-adjusted Charlson comorbidity index; **CRIF/ORIF**, closed or open reduction and internal fixation; **HA**, hemiarthroplasty; **THA**, total hip arthroplasty.
